# Supplementary material for: Spatially resolved gene regulatory and disease-related vulnerability map of the adult Macaque cortex
Source: Nat Commun. 2022 Nov 8;13:6747. doi: 10.1038/s41467-022-34413-3 (PMC9643508; doi:10.1038/s41467-022-34413-3)
Supplement: Supplementary file 1 — Supplementary Information [file 41467_2022_34413_MOESM1_ESM.pdf]

Spatially resolved gene regulatory and disease-related vulnerability map of the adult Macaque cortex

Supplementary Information

Supplementary Fig.1

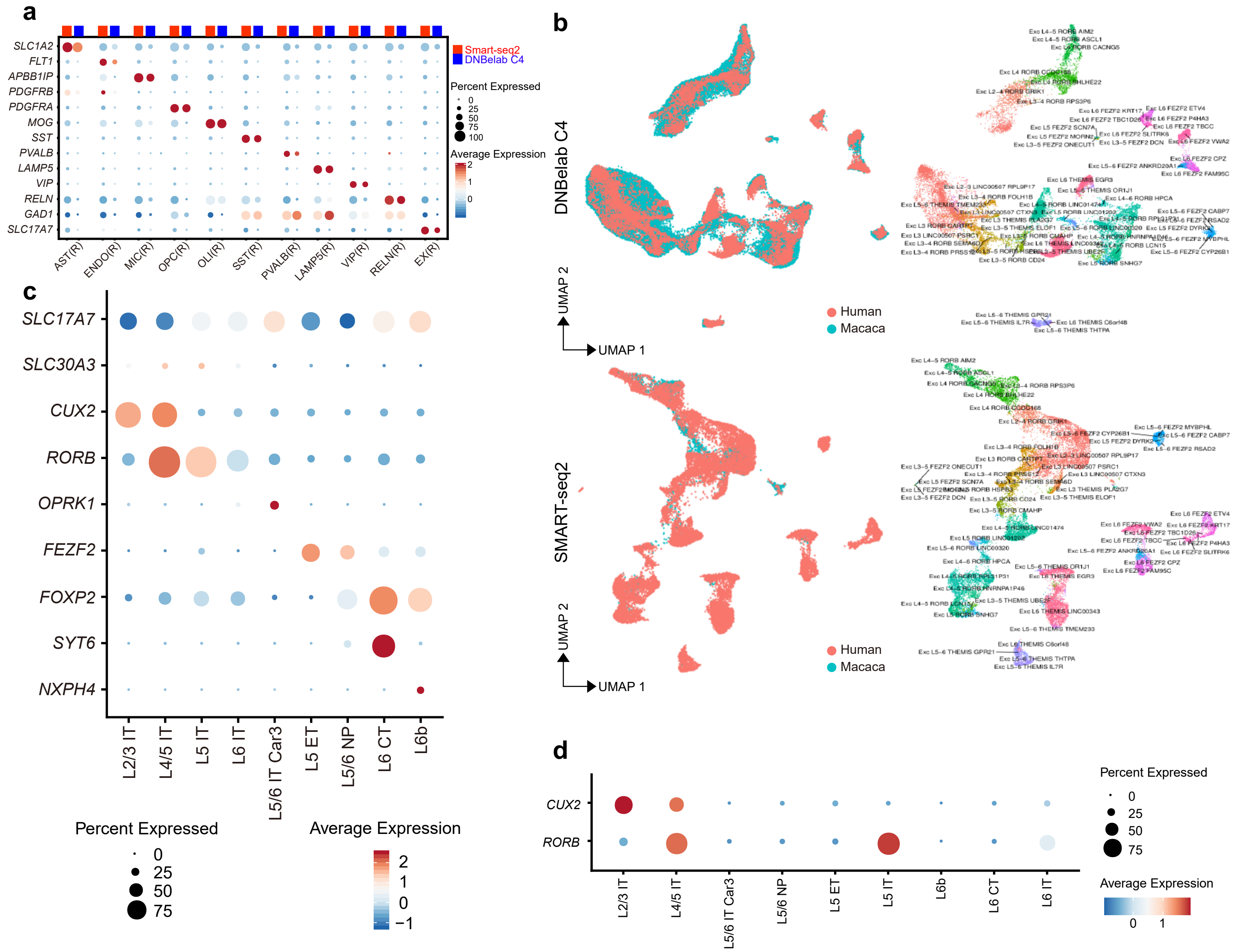

Supplementary Fig. 1 Cell type annotation of snRNA-seq data. a Dot plots of expression values for neuronal and non-neuronal cell type-specific marker genes in Smart-seq2 and DNBelab C4 snRNA-seq data. **b** Integration of DNBelab C4 (top) and Smart-seq2 (bottom) snRNA-seq data of excitatory neurons from macaque with single-nucleus transcriptomes of human cortex[1,2] (left) (Allen Cell Types Database-Human Multiple Cortical Areas,https://portal.brain-map.org/atlases-and-data/rnaseq/human-multiple-cortical-areas-smart-seq), and cluster labels of human excitatory neurons were indicated (right). **c** Expression of marker genes for mouse excitatory cell classes[3,4] in macaque excitatory neuron subtypes. **d** Dot plots showing layer marker of L2-4 (CUX2) and L3-5 (RORB) are co-expressed in L4/5 IT of M1.

Source data are provided as a Source Data file.

Supplementary Fig.2

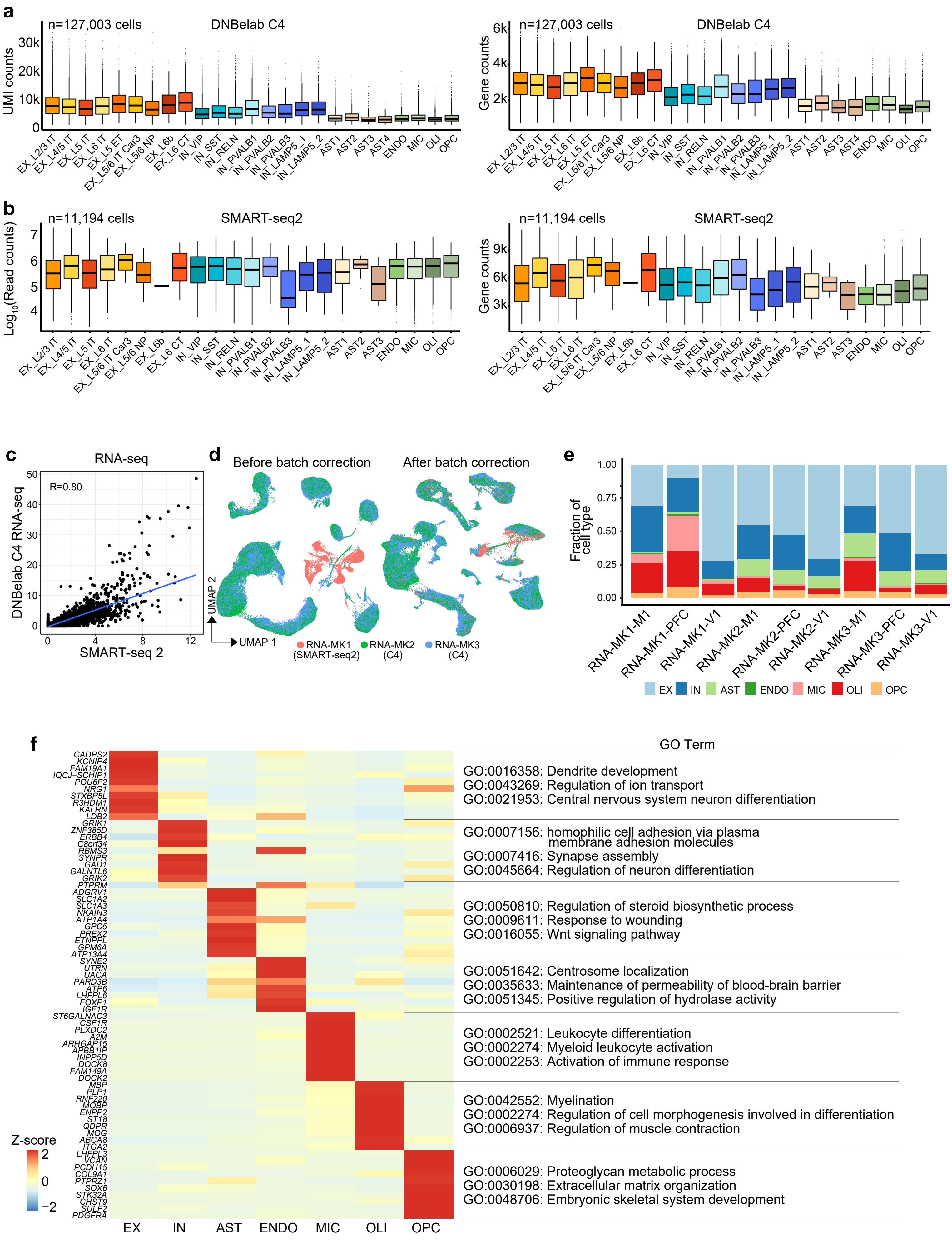

Supplementary Fig 2 Quality assessment of snRNA-seq data. a Box plot of unique molecular identifiers counts (UMI) (left) and detected gene number (right) of DNBelab-C4 cells in each snRNA-seq cell type. Boxes extend from the 25th to 75th percentiles, line in the middle of the box is plotted at the median. Whiskers =min and max. **b** Box plot of reads count (left) and detected gene number (right) of Smart-seq2 cells in each snRNA-seq cell type. Boxes extend from the 25th to 75th percentiles, line in the middle of the box is plotted at the median. Whiskers =min and max. **c** Correlation between Smart-seq2 data and DNBelab C4 snRNA-seq data, R value was calculated by Pearson correlation. **d** UMAP projections of all primary snRNA-seq cells before (left) and after (right) batch correction colored by individual donor (the library preparation methods are indicated). **e** Proportion of each major cell type in each cortical region of each individual donor from snRNA-seq cells. **f** Heatmap displaying the differentially expressed genes of major cell classes in snRNA-seq data. Specific genes related to each cell type are highlighted with enriched gene ontology terms.
Source data are provided as a Source Data file.

Supplementary Fig.3

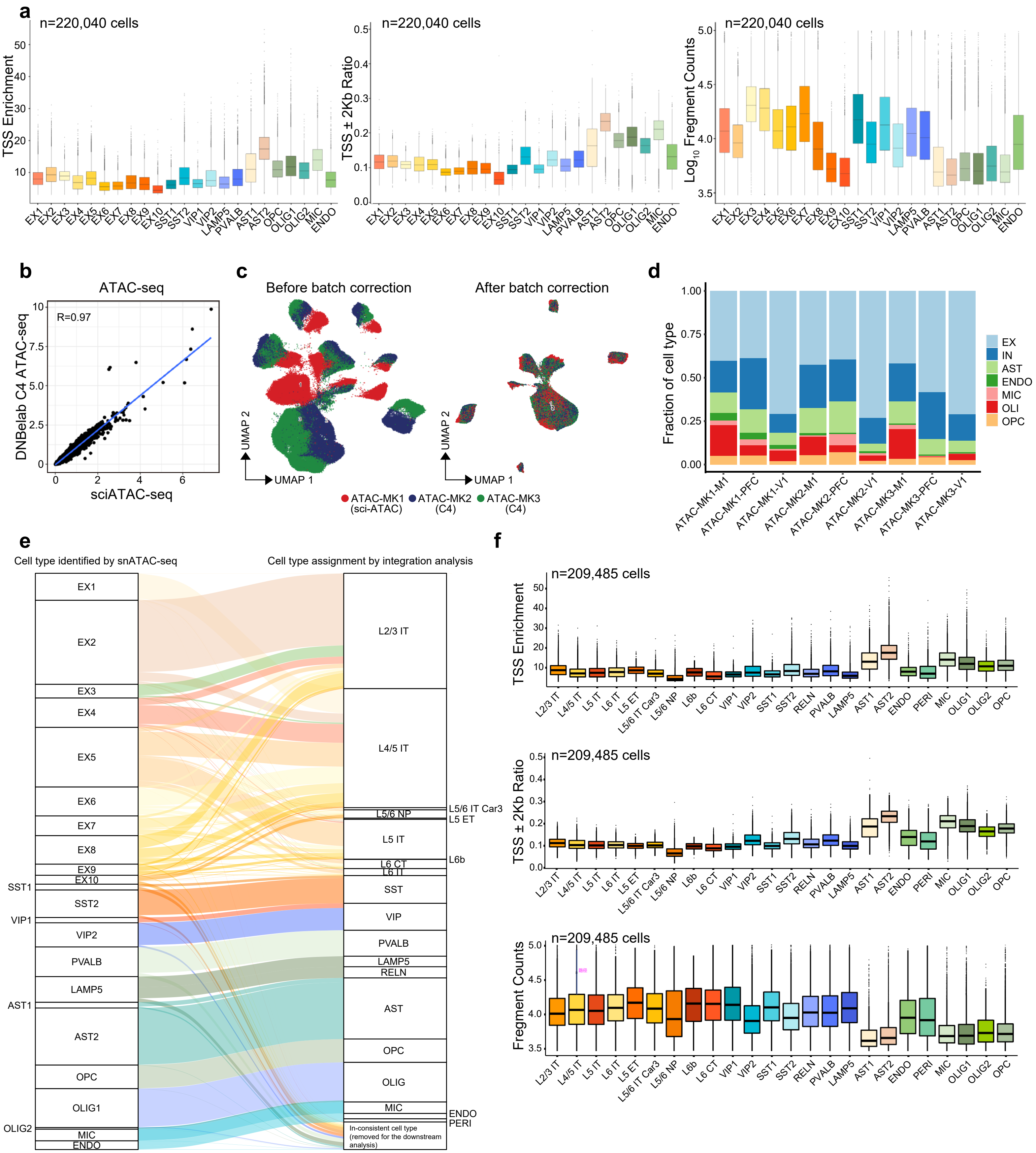

Supplementary Fig. 3 Quality assessment of snATAC-seq data. a Box plot of TSS enrichment: average accessibility of the TSS +/- 50 bp region/ average accessibility of the TSS flanking positions (+/- 1900 – 2000 bp) (left), TSS ± 2 kb ratio: the proportion of peaks within 2 kb from the gene TSS site (middle) and fragment counts (right) of snATAC-seq cells in each snATAC-seq cluster. Boxes extend from the 25th to 75th percentiles, line in the middle of the box is plotted at the median. Whiskers =min and max. **b** Correlation between sciATAC-seq data and DNBelab C4 snATAC-seq data, R value was calculated by Pearson correlation. **c** UMAP projections of all primary snATAC-seq cells before (left) and after (right) batch correction colored by individual donor (the library preparation methods are indicated). **d** Proportion of each major cell type in each cortical region of each individual donor from snATAC-seq cells. **e** Alluvial plot depicting mappings between cell type of snATAC-seq clusters and cell type assigned by integration analysis with snRNA-seq. **f** Box plot of TSS enrichment: average accessibility of the TSS +/- 50 bp region/ average accessibility of the TSS flanking positions (+/- 1900 – 2000 bp) (top), TSS ± 2 kb ratio: the proportion of peaks within 2 kb from the gene TSS site (middle) and fragment counts (bottom) of snATAC-seq cells with consistent cell type identified by snATAC-seq and integration analysis. Boxes extend from the 25th to 75th percentiles, line in the middle of the box is plotted at the median. Whiskers =min and max.

Source data are provided as a Source Data file.

Supplementary Fig.4

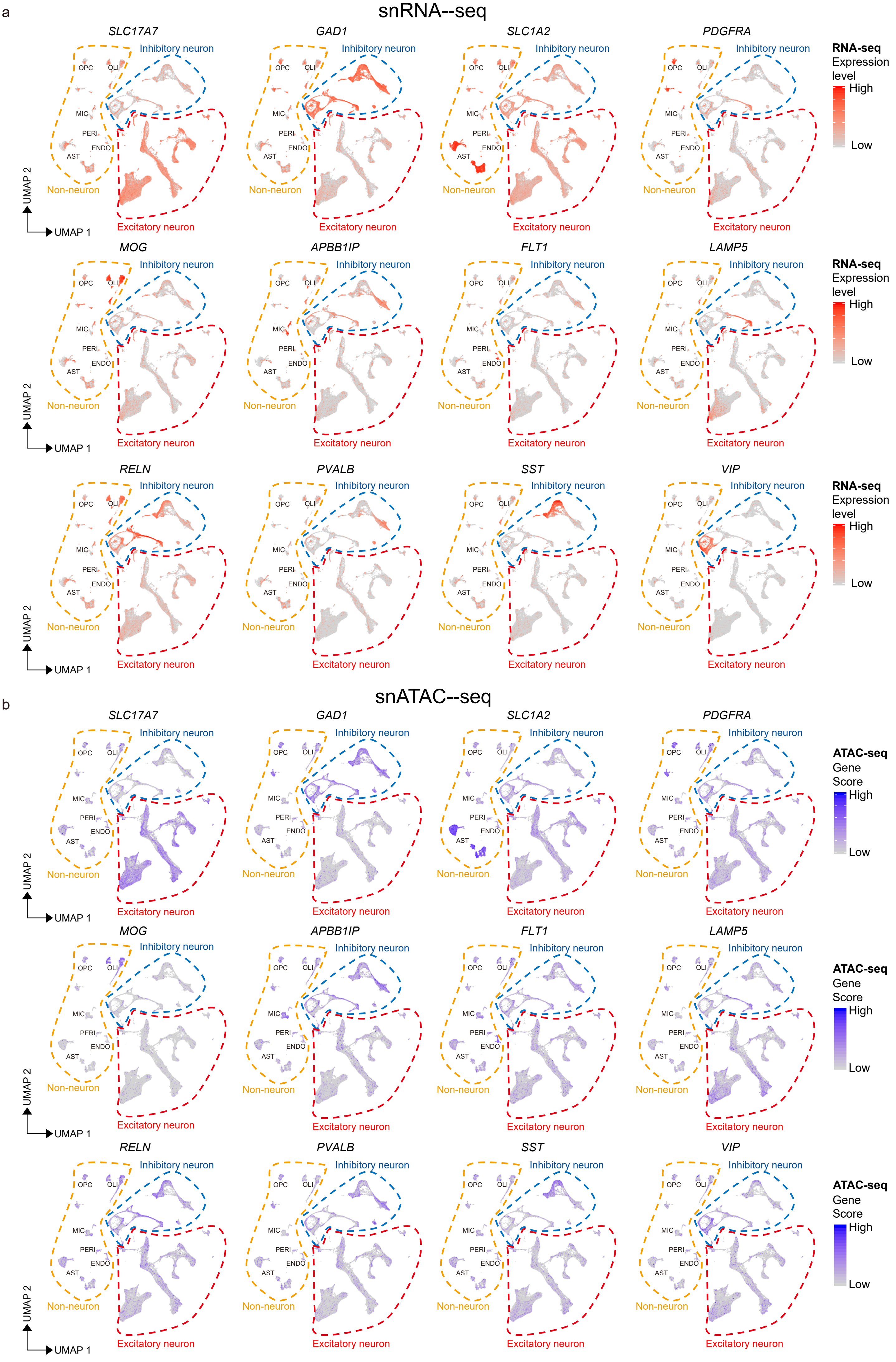

Supplementary Fig. 4 Congruent cell type identifications in snRNA-seq cells and snATAC-seq cells. a Expression levels of cell type-specific marker genes in snRNA-seq cells visualized by UMAP. **b** Gene activity score of cell type-specific marker genes in snATAC-seq cells visualized by UMAP.
Source data are provided as a Source Data file.

Supplementary Fig.5

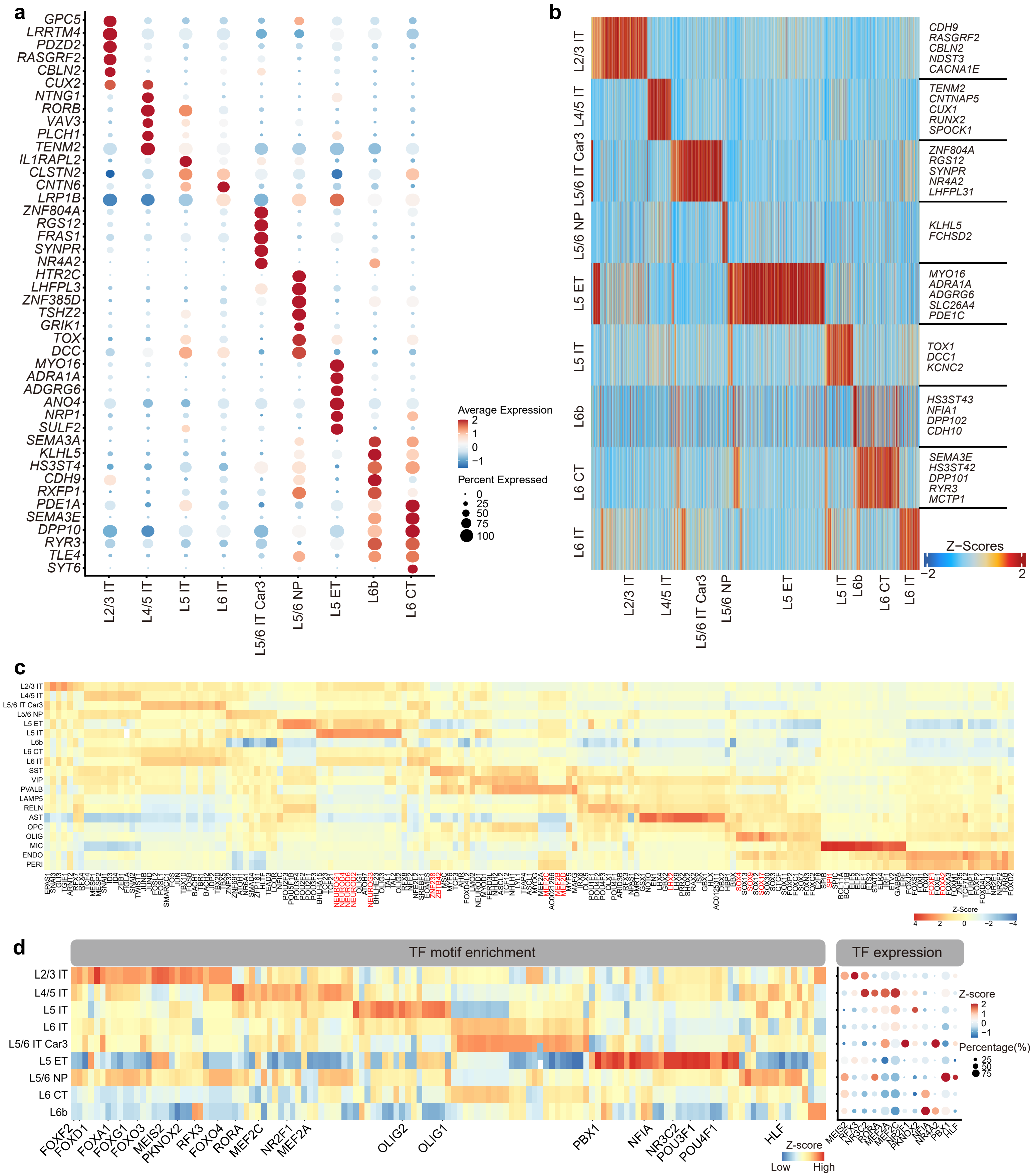

Supplementary Fig. 5 Chromatin accessibility and gene expression of excitatory neurons subtypes.a Dot plots showing expression of marker genes across macaque excitatory neuron subtypes of snRNA-seq. **b** Heatmap showing the differentially accessible peaks enriched across macaque excitatory neuron subtypes of snATAC-seq. **c** Heatmap showing the top 15 transcription factor (TF) binding motifs enriched at each snATAC-seq cell type of Fig. 1g. **d** Heatmap showing the transcription factors (TF) that binding motifs (left) and differential gene expression (right) are enriched in corresponding subtypes of excitatory neuron. **Source data are provided as a Source Data file**.

Supplementary Fig.6

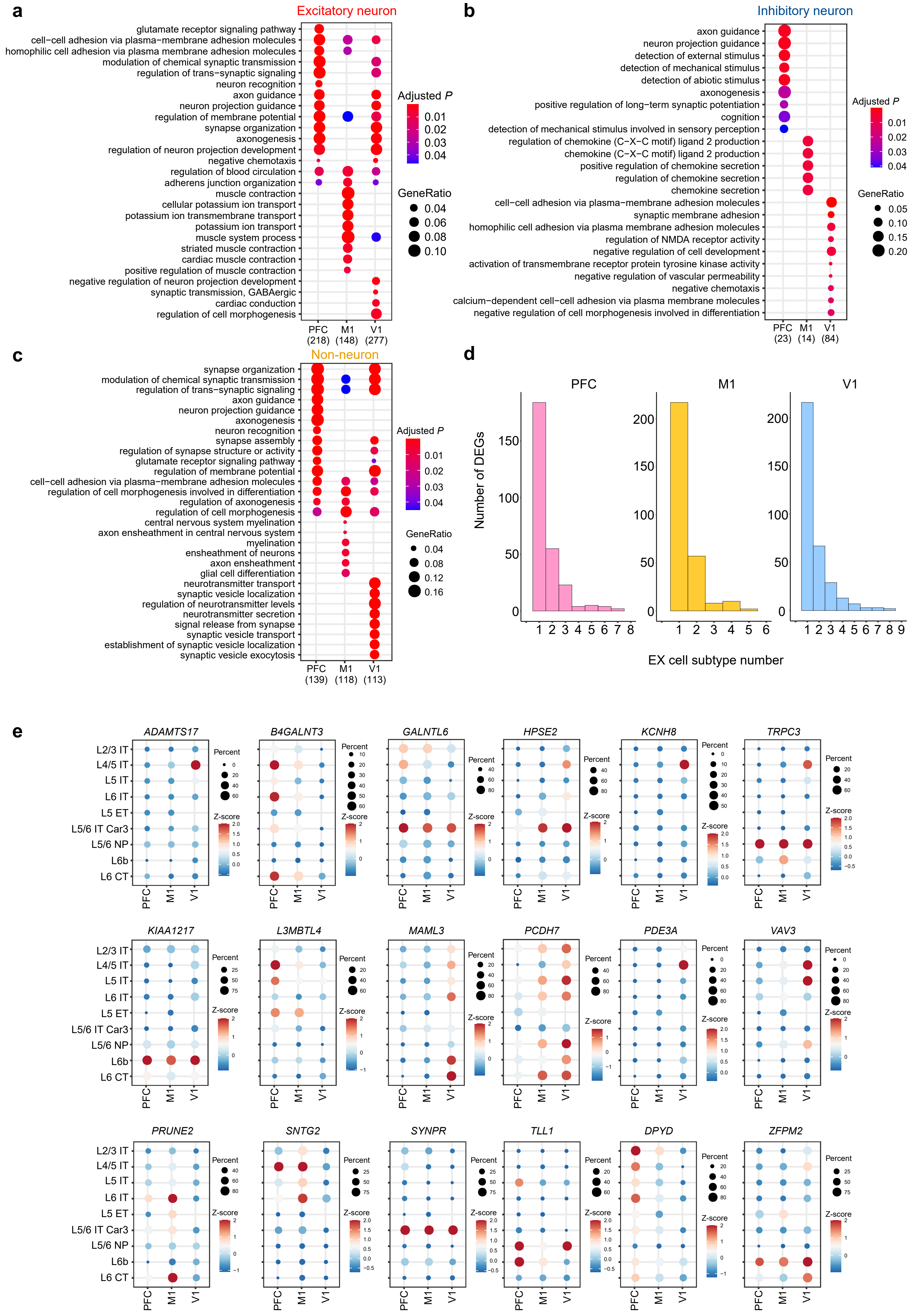

Supplementary Fig. 6 Areal differences within excitatory neurons. a Gene ontology terms enriched among differentially expressed genes in PFC, M1 and V1 of excitatory neuron subtypes. A one-sided hypergeometric test and Bonferroni correction was performed for gene set overlap significance. **b** Gene ontology terms enriched among differentially expressed genes in PFC, M1 and V1 of inhibitory neuron subtypes. A one-sided hypergeometric test and Bonferroni correction was performed for gene set overlap significance. **c** Gene ontology terms enriched among differentially expressed genes in PFC, M1 and V1 of non-neuronal cell types. A one-sided hypergeometric test and Bonferroni correction was performed for gene set overlap significance. **d** Histogram of areal DEGs appeared in one through nine EX subtypes. **e** Dot plots of selected differentially expressed genes in PFC, M1 and V1 of excitatory neuron subtypes.
Source data are provided as a Source Data file.

Supplementary Fig.7

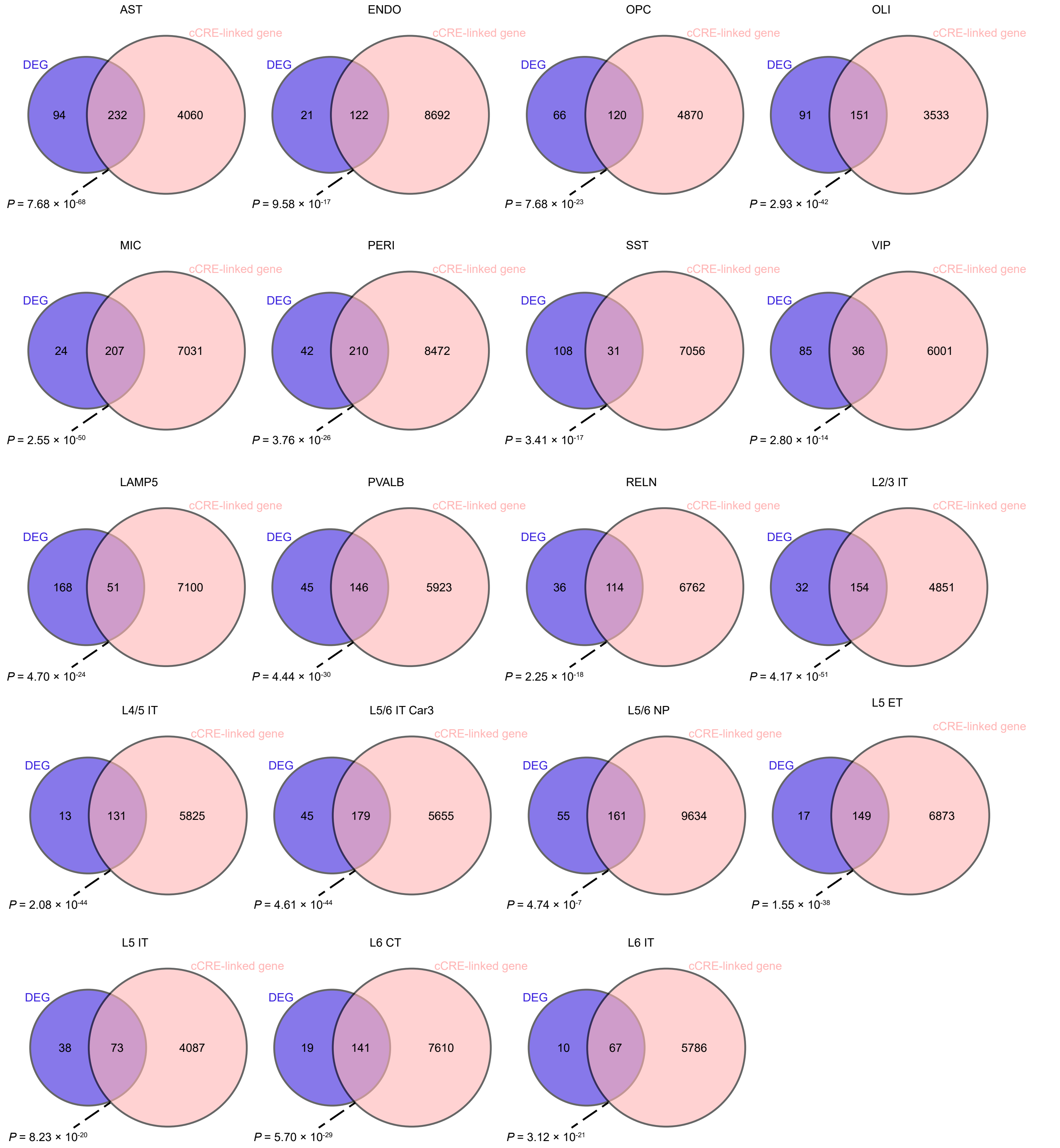

Supplementary Fig. 7 Linking *cis*-regulatory elements to cell type specific genes. Venn diagrams showing the overlaps between CRE-targeted genes and differentially expressed genes (DEGs) in that cell type/subtype. A one-sided hypergeometric test was performed for gene set overlap significance.
Source data are provided as a Source Data file.

Supplementary Fig.8

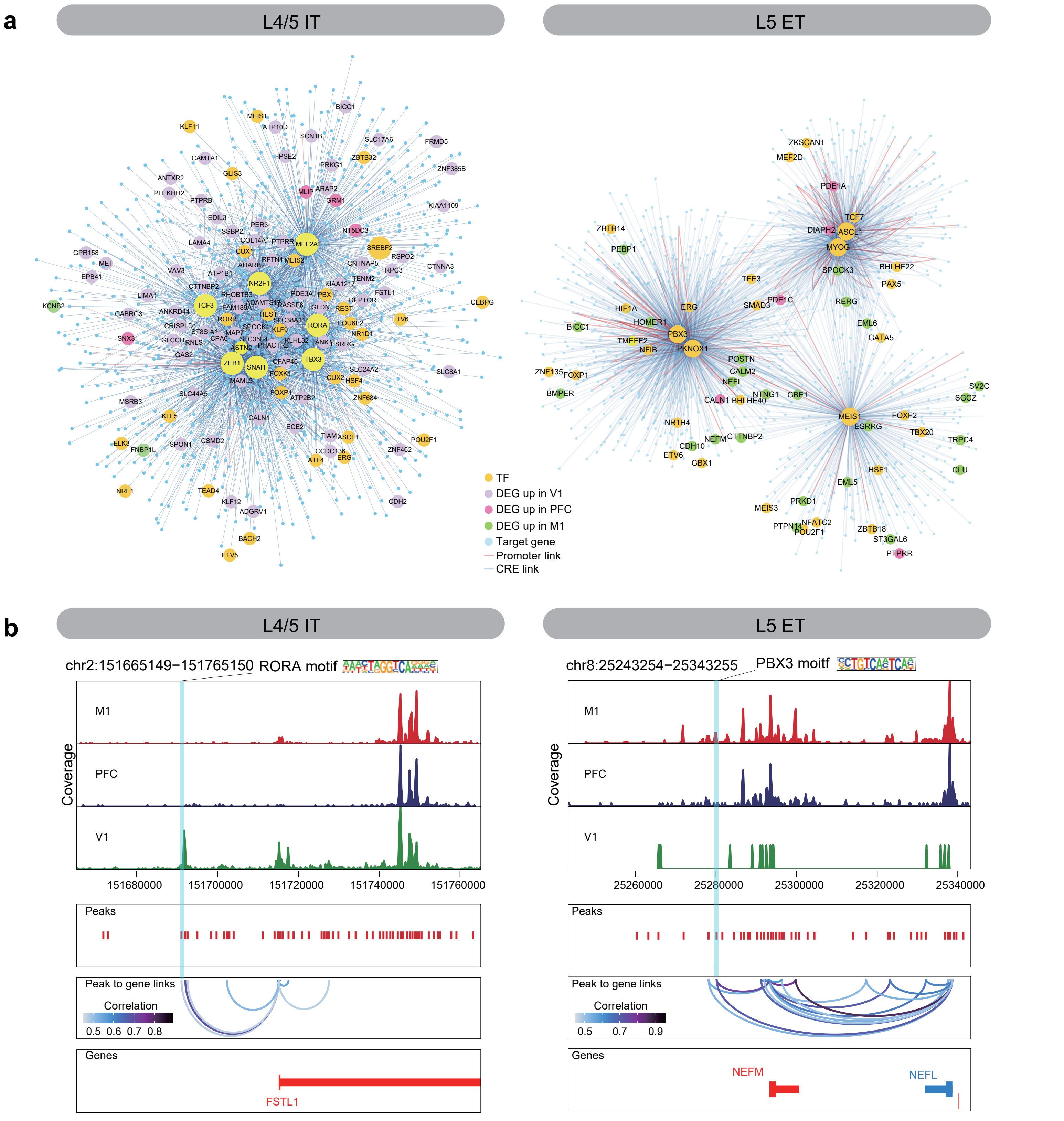

Supplementary Fig. 8 Regulatory network of region-specific genes. a TF regulatory networks showing the predicted candidate target genes for transcription factors RORA, NR2F1, ZEB1, SNAI1, TBX3, MEF2A and TCF3 in L4/5 IT type excitatory neuron (left) and the predicted candidate target genes for transcription factors MEIS1, PBX3, ASCL1, MYOG and PKNOX1 in L5 ET type excitatory neuron (right). **b** Inferences of predictive TFs binding motif and distal links to V1-specific gene *FSTL1* in L4/5 IT cells and M1-specific gene *NEFM* and *NEFL* in L5 ET cells.

Source data are provided as a Source Data file.

Supplementary Fig.9

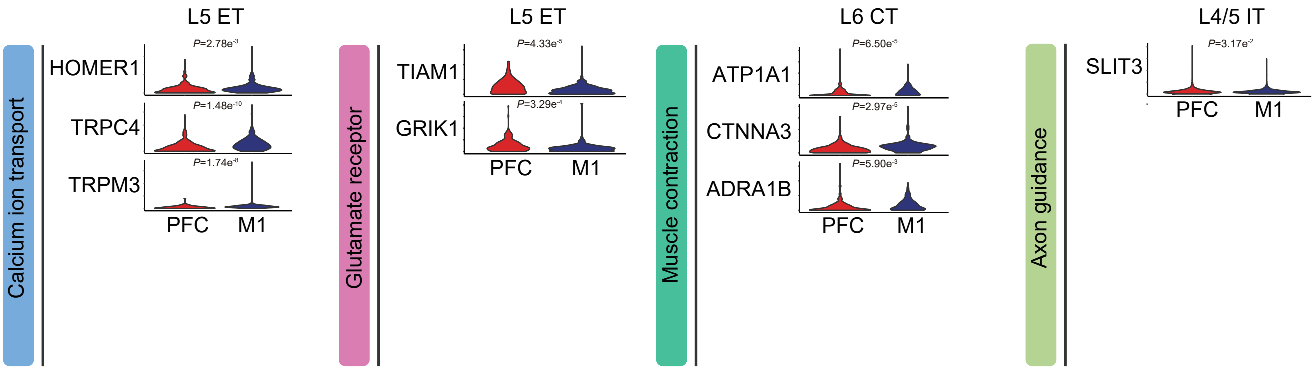

Supplementary Fig. 9. Specialized transcriptomics of excitatory neuron between macaque prefrontal cortex and motor cortex.
Violin plots indicating significantly upregulated gene score of selected DEGs (Fig. 3b) between PFC and M1 of L5 ET, L6 CT, L4/5 IT and L6 IT type excitatory neuron, respectively. Unpaired two-samples one-sided Wilcoxon test and Bonferroni correction was used to perform inter-regional gene score comparison.
Source data are provided as a Source Data file.

Supplementary Fig.10

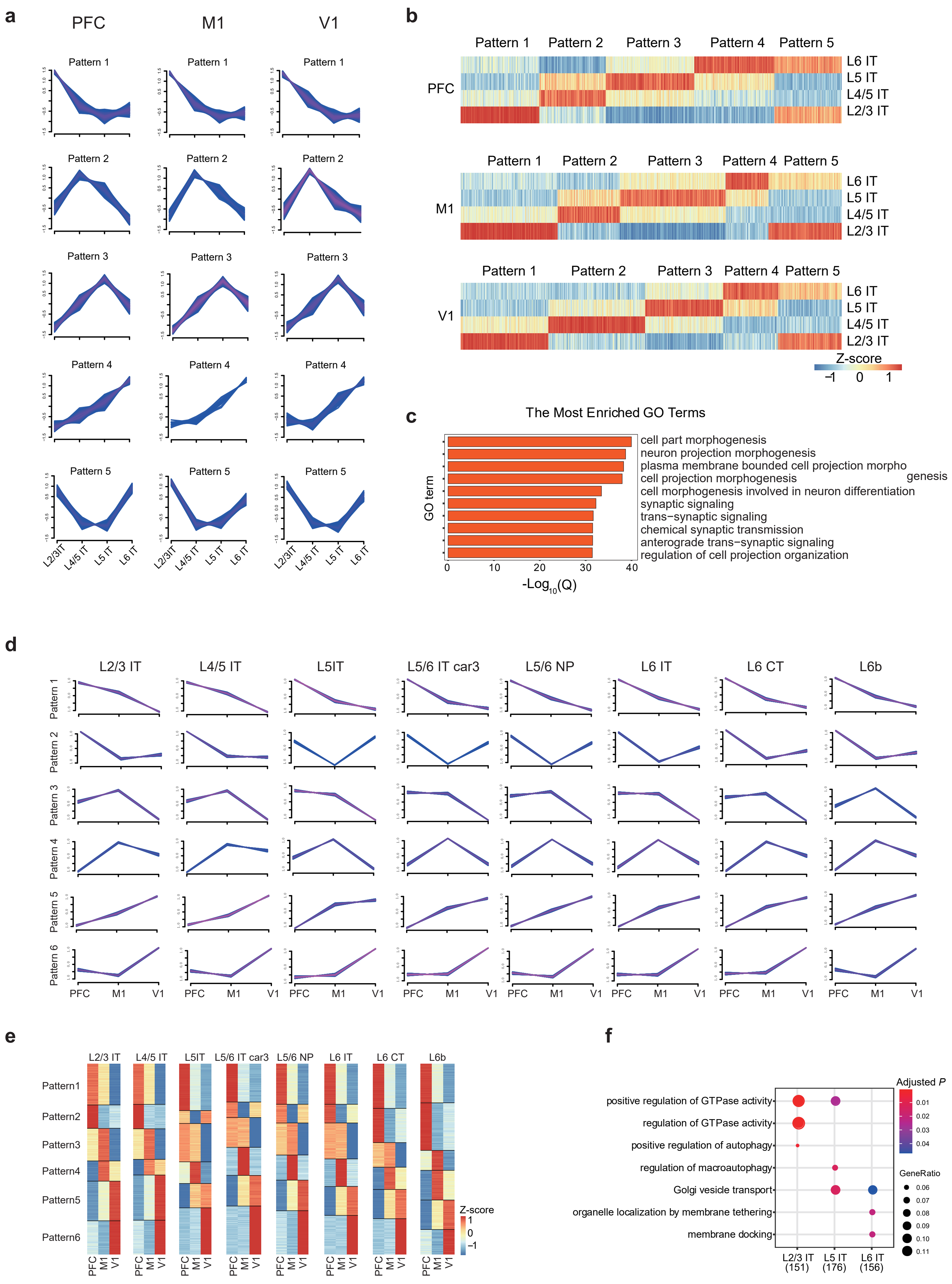

Supplementary Fig. 10. Gradient gene expression pattern of excitatory neurons.
a Gradient genes expression patterns across L2/3 IT, L4/5 IT, L5 IT and L6 IT by Mfuzz in PFC, M1 and V1 (probability of genes matching the pattern > 0.5). **b** Heatmap showing the genes from a) and filtered by maximum expression > 1. **c** Gene ontology terms enriched among genes with distinct expression pattern between PFC, M1 and V1. **d** Gradient gene expression patterns across PFC, M1 and V1 by Mfuzz in excitatory neuron subtypes (probability of genes matching the pattern > 0.5). **e** Heatmap showing the genes from d) and filtered by maximum expression > 1. **f** Gene ontology terms enriched among genes in pattern 1 of L2/3 IT, L5 IT and L6 IT types. A one-sided hypergeometric test and Bonferroni correction was performed for gene set overlap significance.
Source data are provided as a Source Data file.

Supplementary Fig.11

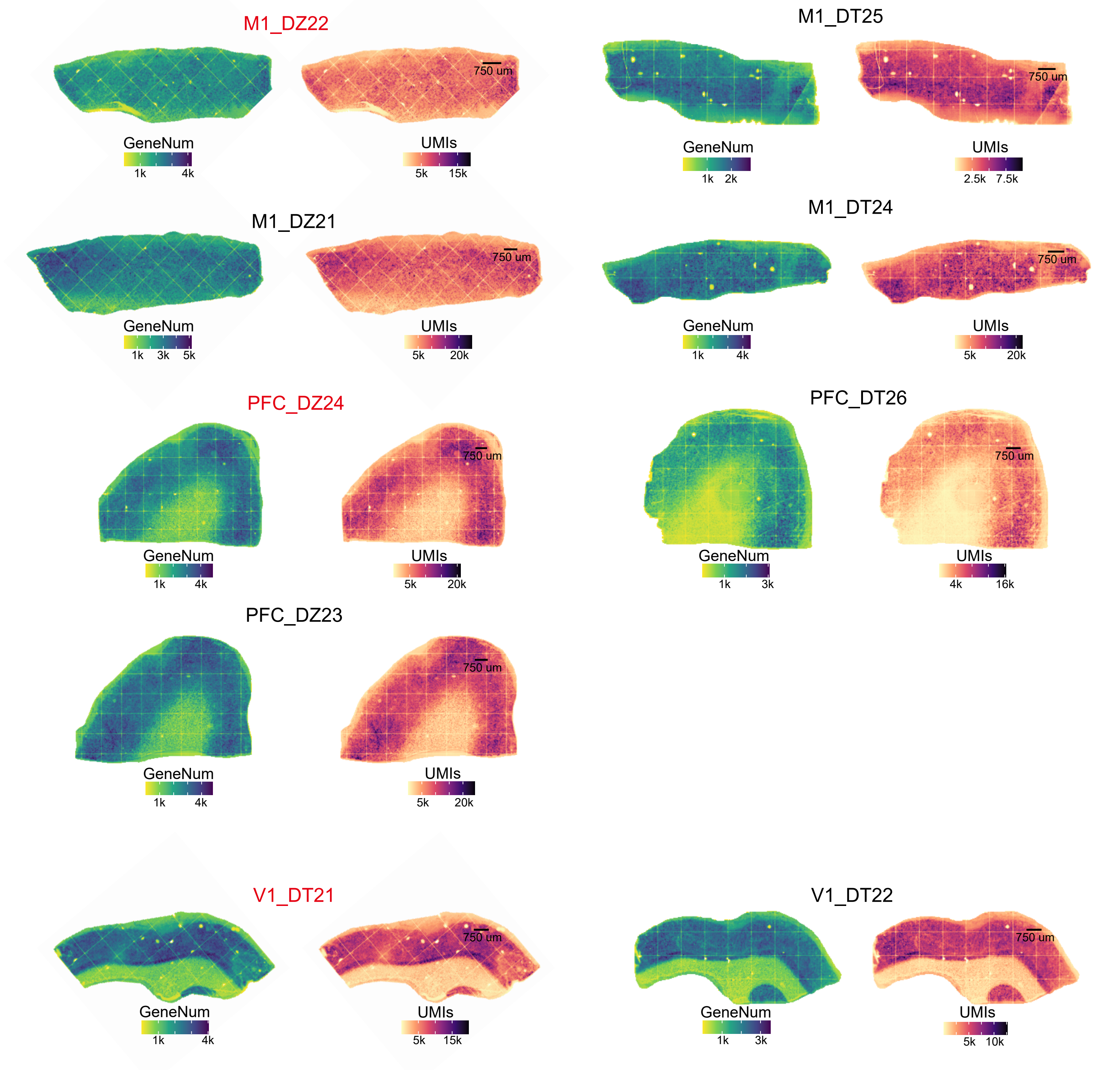

Supplementary Fig. 11. Detected gene number and UMI counts of Stereo-seq sections. Spatial distribution of detected genes number and UMI counts per 37.5 μm bin in Stereo-seq sections from PFC, M1 and V1. Section numbers used for representative micrographs in Figure 4 and Supplementary Figure 12 were red color coded.

Source data are provided as a Source Data file.

Supplementary Fig.12

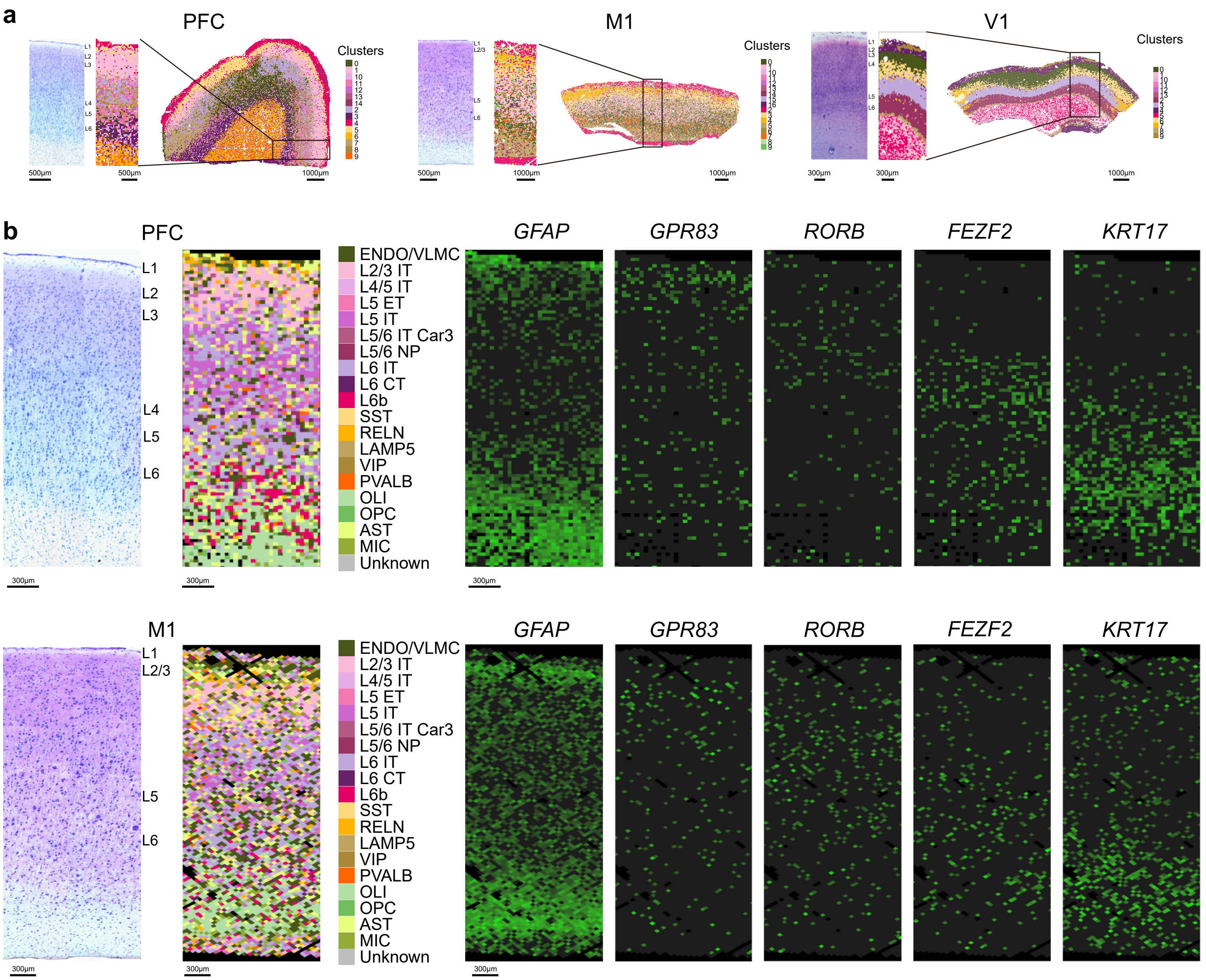

Supplementary Fig. 12. Unsupervised clustering and cell type annotation of Stereo-seq sections. a Nissl staining of adjacent section to stereo-seq section and unsupervised clustering of Stereo-seq spots (37.5 μm bins) of PFC, M1 and V1. **b** Nissl staining of adjacent section to stereo-seq section of PFC (top left) or M1 (bottom left), cell type annotation of stereo-seq spots (37.5 μm bins) by snRNA-seq data (middle), and layer enrichment of known layer-marker genes in the same stereo-seq section (right). Experiments have been performed independently in 3 slices for PFC, 4 slices for M1 and 2 slices for V1.
Source data are provided as a Source Data file.

Supplementary Fig.13

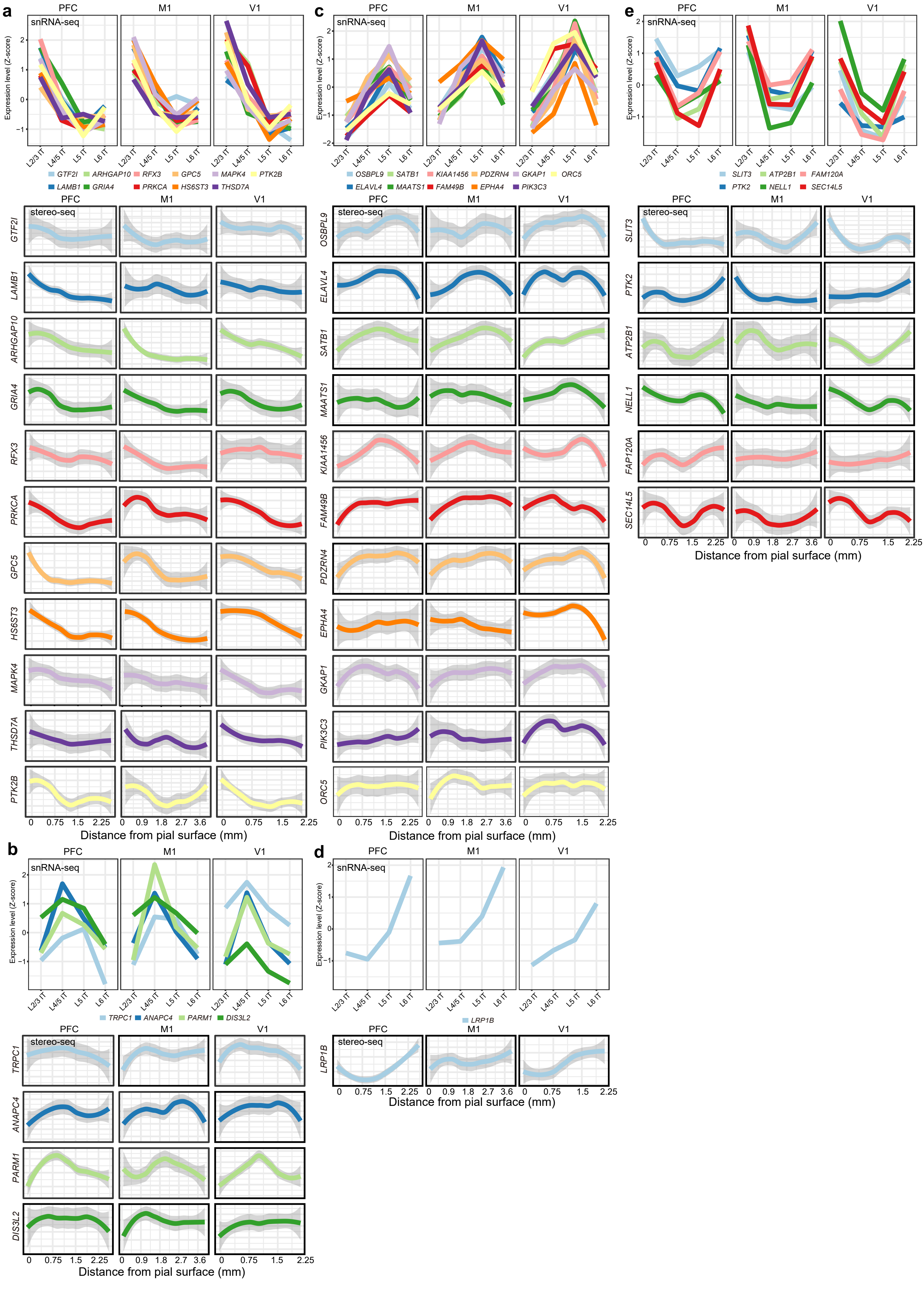

Supplementary Fig. 13. Gradient expressed genes in excitatory neuron of Stereo-seq sections.

Genes showed congruent gradient expression pattern between PFC, M1 and V1 of snRNA-seq cells also exert consensus expression pattern in PFC, M1 and V1 of Stereo-seq. a-e corresponding to pattern 1-5 in Supplementary Fig. 9a,b. The grey shaded error bands in a-e shows the mean ± S.E.M.

Source data are provided as a Source Data file.

Supplementary Fig.14

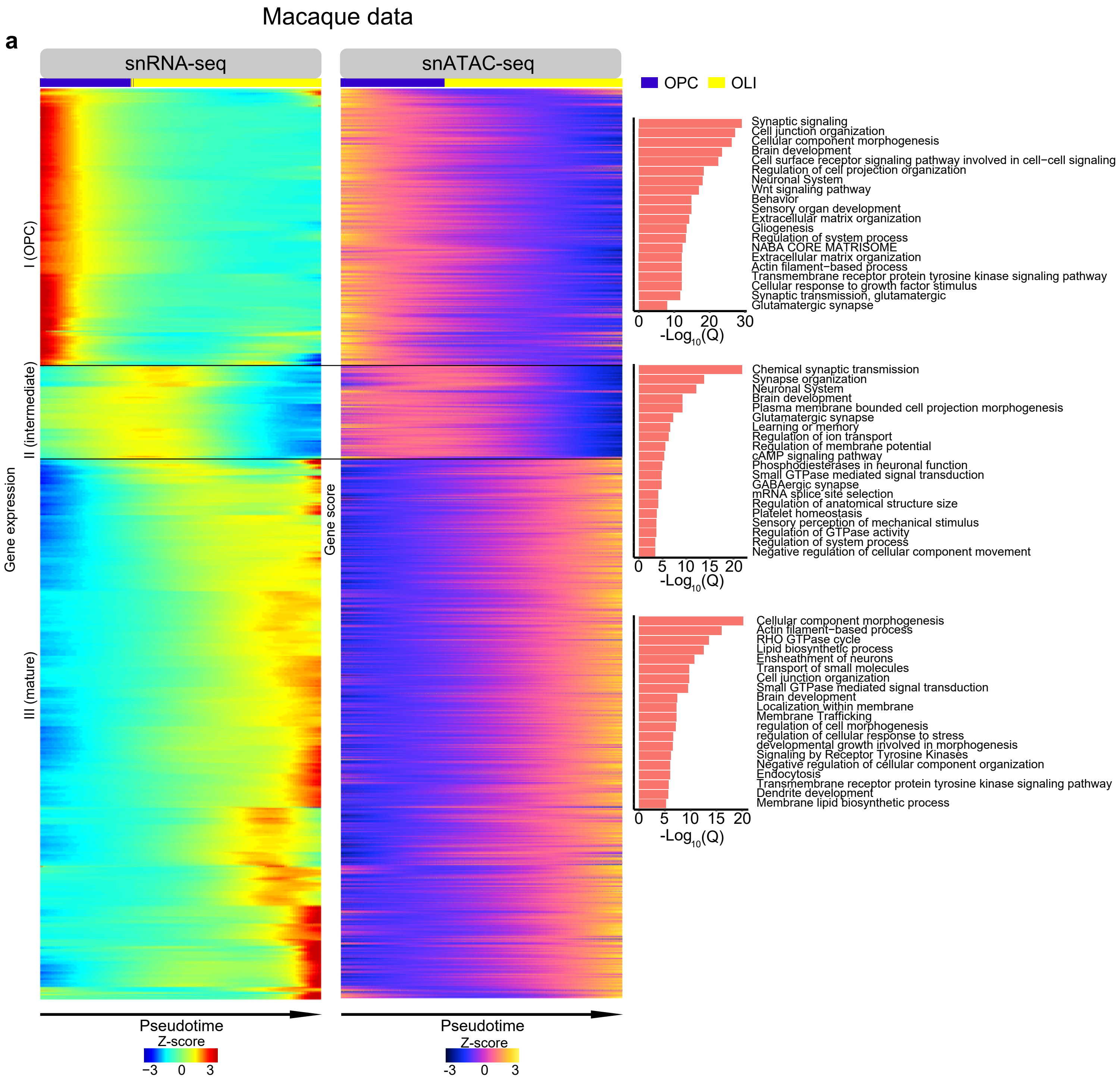

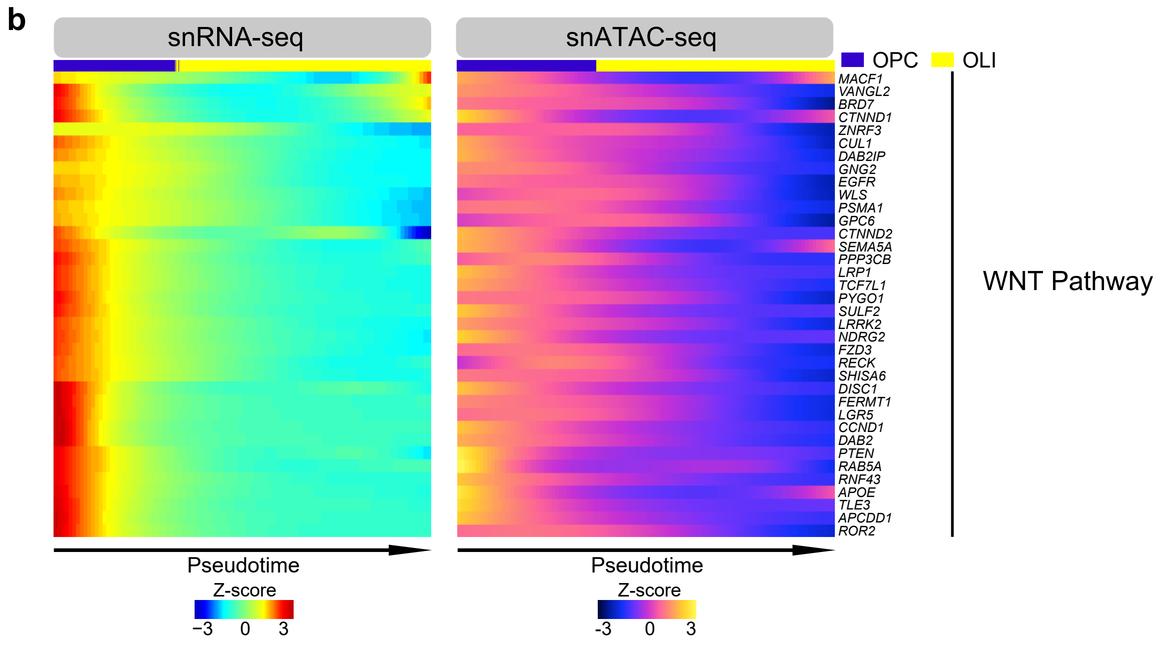

Supplementary Fig. 14 Well-concordance gene expression-gene activity score pairs along macaque OLI lineage trajectory. a Well-concordance gene expression (left)-gene activity scores (middle) pairs and ontologies terms (right) along macaque OLI pseudotime trajectory (Two-sided Pearson correlation coefficient $R > 0.2$, $P < 0.01$). **b** Well-concordance gene expression (left)-gene activity score (right) pairs of genes involved in Wnt signaling pathway along macaque OLI pseudotime trajectory.
Source data are provided as a Source Data file.

Supplementary Fig.15

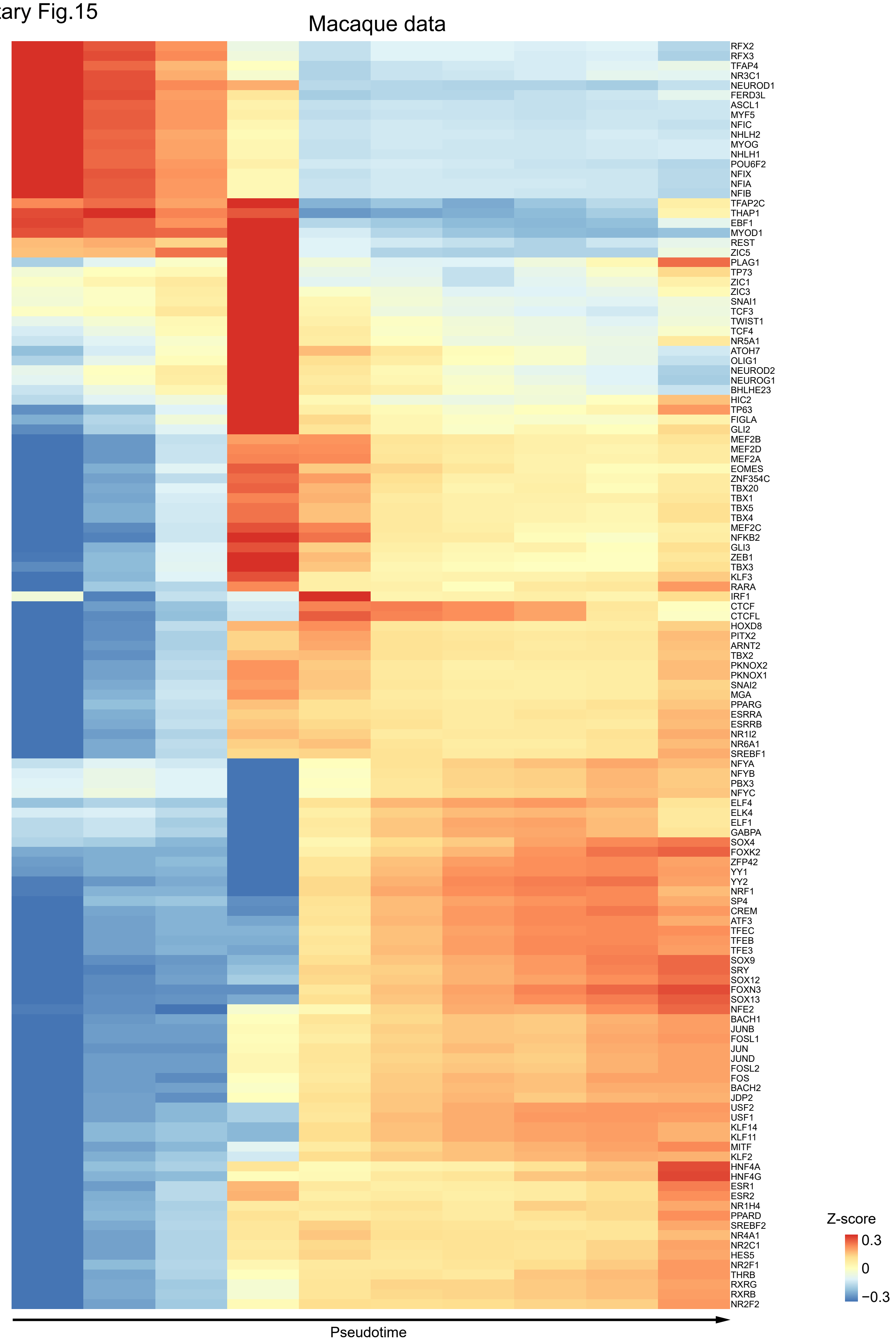

Supplementary Fig. 15 TF motif enrichment along macaque OLI lineage trajectory. Heatmap ordering of average TF binding motif bias-corrected deviations for 129 most variable TFs along macaque OLI pseudotime.

Source data are provided as a Source Data file.

Supplementary Fig.16

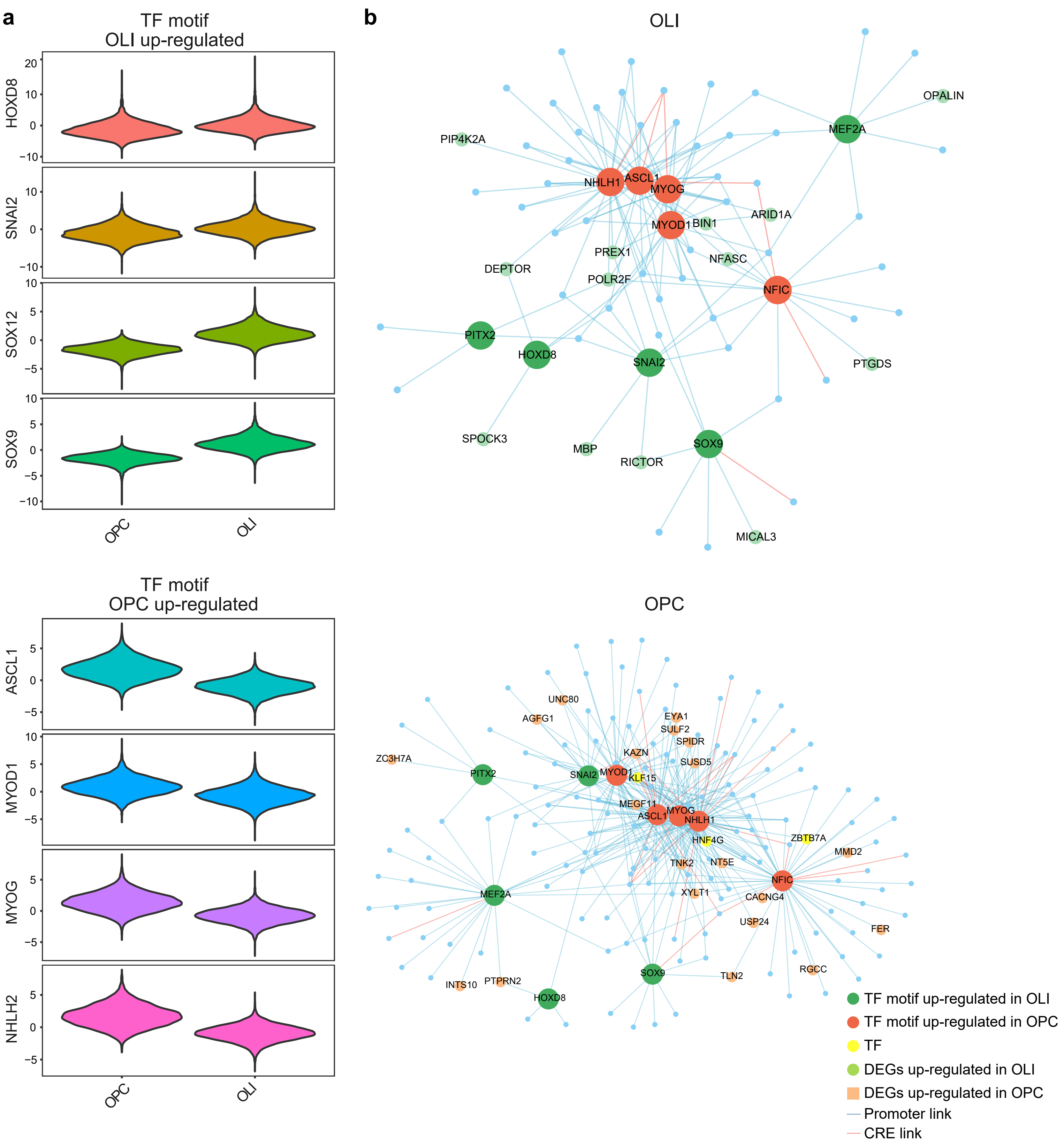

Supplementary Fig. 16 TF regulatory networks of OPC and OLI. a Violin plots of motif enrichment of selected TFs that had significant upregulated in snATAC-seq cells of OPC (top) and OLI (bottom). **b** TF regulatory networks of OPC (top) and OLI (bottom) showing the predicted candidate target genes for transcription factors in a).

Source data are provided as a Source Data file.

Supplementary Fig.17

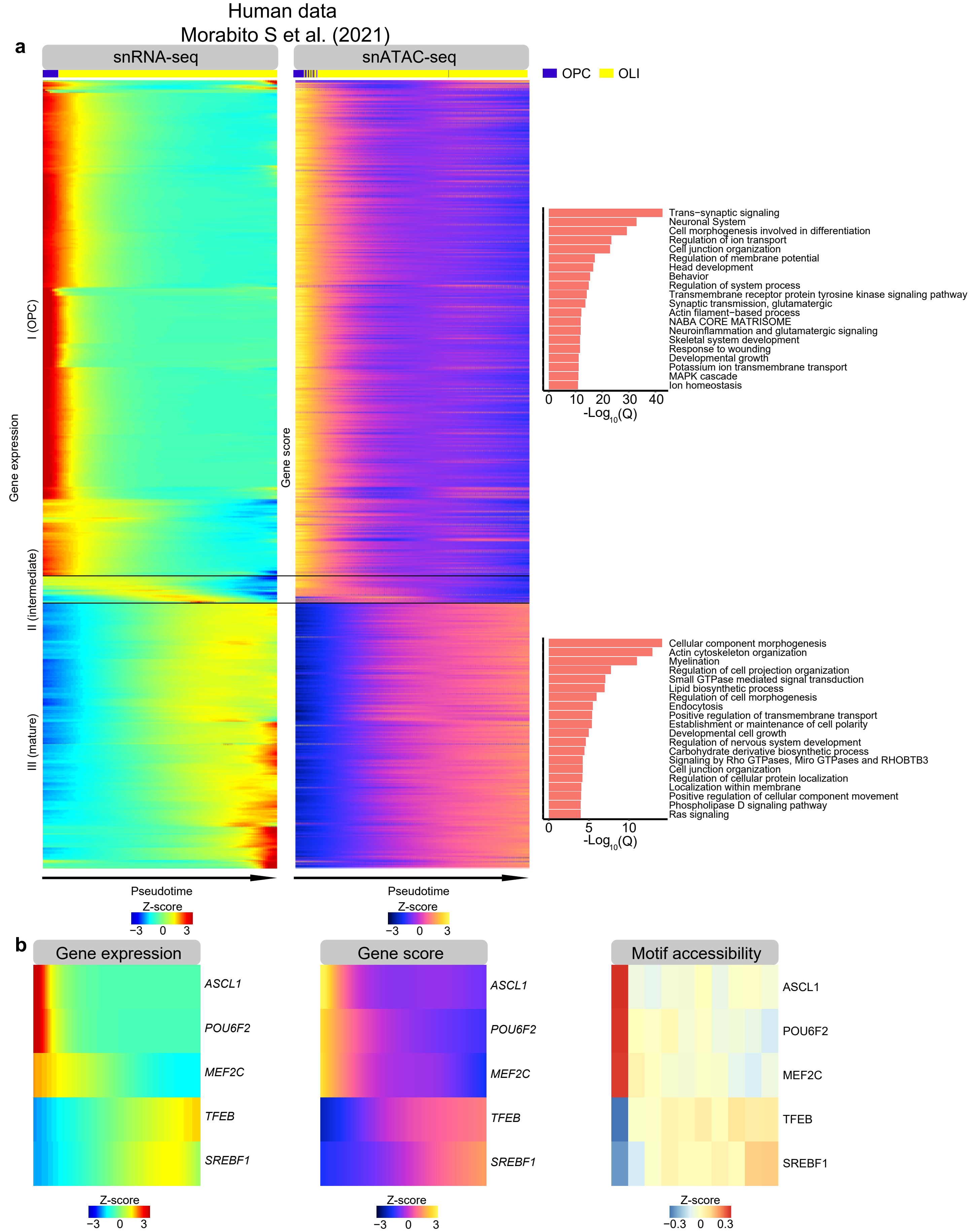

Supplementary Fig. 17 Well-concordance gene expression-gene activity score pairs along human OLI lineage trajectory. a Well-concordance gene expression (left)-gene activity scores (middle) pairs and ontologies terms (right) along human OLI[5] pseudotime trajectory (Two-sided Pearson correlation coefficient $R > 0.2$, $P < 0.01$). **b** Gene expression (left), gene activity score (middle) and motif enrichment (right) of 5 TFs across human OLI[5] pseudotime trajectory.

Source data are provided as a Source Data file.

Supplementary Fig.18

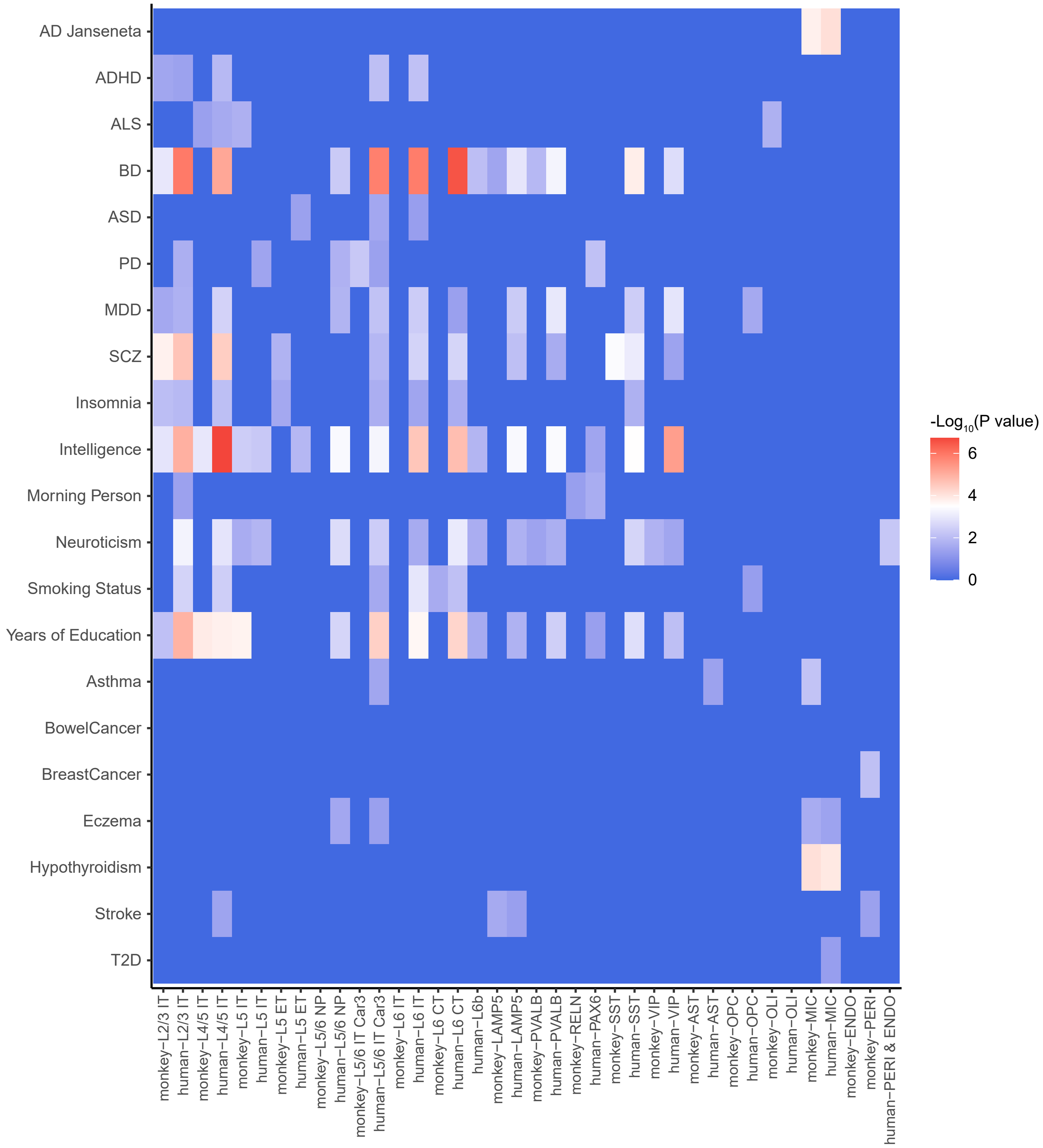

Monkey： Our data Human： Morabito S et al. (2021)

Supplementary Fig. 18 Cell type enrichment of heritability in macaque and human cortex.
Heatmap showing the enrichments of heritability for human traits among the neuronal and non-neuronal cell types from snATAC-seq data of macaque and human[5] prefrontal cortex. P values were derived from LDSC enrichment tests. AD, Alzheimer's disease; ADHD, attention deficit hyperactivity disorder; ASD, autism spectrum disorder; BD, bipolar disorder; ALS, amyotrophic lateral sclerosis; MDD, major depression disorders; SCZ, schizophrenia, T2D, type 2 diabetes.
Source data are provided as a Source Data file.

Supplementary References

1 Hodge, R. D. *et al.* Conserved cell types with divergent features in human versus mouse cortex. *Nature* **573**, 61-68, doi:10.1038/s41586-019-1506-7 (2019).

2 Network, B. I. C. C. A multimodal cell census and atlas of the mammalian primary motor cortex. *Nature* **598**, 86-102, doi:10.1038/s41586-021-03950-0 (2021).

3 Yao, Z. *et al.* A taxonomy of transcriptomic cell types across the isocortex and hippocampal formation. *Cell*, doi:10.1016/j.cell.2021.04.021 (2021).

4 Yao, Z. *et al.* A transcriptomic and epigenomic cell atlas of the mouse primary motor cortex. *Nature* **598**, 103-110, doi:10.1038/s41586-021-03500-8 (2021).

5 Morabito, S. *et al.* Single-nucleus chromatin accessibility and transcriptomic characterization of Alzheimer's disease. *Nat Genet* **53**, 1143-1155, doi:10.1038/s41588-021-00894-z (2021).
