# Supplementary material for: Spatially resolved gene regulatory and disease-related vulnerability map of the adult Macaque cortex
Source: Nat Commun. 2022 Nov 8;13:6747. doi: 10.1038/s41467-022-34413-3 (PMC9643508; doi:10.1038/s41467-022-34413-3)
Supplement: Supplementary file 2 — Description of Additional Supplementary Files [file 41467_2022_34413_MOESM2_ESM.pdf]

Description of Additional Supplementary Files

File Name: Supplementary Data 1
Description: Sample annotation and quality information of snRNA-seq and snATAC-seq data.

File Name: Supplementary Data 2
Description: Differentially expressed genes among snRNA-seq cell type.

File Name: Supplementary Data 3
Description: Differentially expressed genes and differentially accessible peaks of layer/projection-defined 9 EX subtypes.

File Name: Supplementary Data 4
Description: Transcription factor (TF) motifs enrichment in each snATAC-seq cell type

File Name: Supplementary Data 5
Description: Differentially expressed genes between PFC, M1 and V1 in each cortical cell type.

File Name: Supplementary Data 6
Description: Differentially accessible peaks between PFC, M1 and V1 in each cortical cell type.

File Name: Supplementary Data 7
Description: Differentially expressed genes between PFC and M1 in excitatory neuron subtypes.

File Name: Supplementary Data 8
Description: Gradient gene expression patterns across L2/3 IT, L4/5 IT, L5 IT and L6 IT by Mfuzz in PFC, M1 and V1.

File Name: Supplementary Data 9
Description: Gradient gene expression patterns across PFC, M1 and V1 by Mfuzz in excitatory neuron subtypes.

File Name: Supplementary Data 10
Description: Differentially expressed genes between PFC, M1 and V1 in excitatory neuron subtypes of snRNA-seq also area-enriched in Stereo-seq sections.

File Name: Supplementary Data 11
Description: Well-concordance set of genes in expression-activity score pairs along oligodendrocyte pseudotime trajectory in macaque monkey and human.

File Name: Supplementary Data 12
Description: Summary of GWAS studies of psychiatric disorders and neurobehavioral traits used for disease risk mapping.
