# Supplementary material for: Spatially resolved gene regulatory and disease-related vulnerability map of the adult Macaque cortex
Source: Nat Commun. 2022 Nov 8;13:6747. doi: 10.1038/s41467-022-34413-3 (PMC9643508; doi:10.1038/s41467-022-34413-3)
Supplement: Supplementary file 6 — Reporting Summary [file 41467_2022_34413_MOESM6_ESM.pdf]

nature portfolio

Corresponding author(s): Xun Xu, Shiping Liu, Longqi Liu, Yong Hou, Hongkui Zeng, Yuyu Niu

Last updated by author(s): Oct 9, 2022

Reporting Summary

Nature Portfolio wishes to improve the reproducibility of the work that we publish. This form provides structure for consistency and transparency in reporting. For further information on Nature Portfolio policies, see our Editorial Policies and the Editorial Policy Checklist.

Statistics

For all statistical analyses, confirm that the following items are present in the figure legend, table legend, main text, or Methods section.

n/a	Confirmed	
☐	☒	The exact sample size (*n*) for each experimental group/condition, given as a discrete number and unit of measurement
☐	☒	A statement on whether measurements were taken from distinct samples or whether the same sample was measured repeatedly
☐	☒	The statistical test(s) used AND whether they are one- or two-sided<br>*Only common tests should be described solely by name; describe more complex techniques in the Methods section.*
☒	☐	A description of all covariates tested
☐	☒	A description of any assumptions or corrections, such as tests of normality and adjustment for multiple comparisons
☐	☒	A full description of the statistical parameters including central tendency (e.g. means) or other basic estimates (e.g. regression coefficient) AND variation (e.g. standard deviation) or associated estimates of uncertainty (e.g. confidence intervals)
☐	☒	For null hypothesis testing, the test statistic (e.g. *F*, *t*, *r*) with confidence intervals, effect sizes, degrees of freedom and *P* value noted<br>*Give P values as exact values whenever suitable.*
☒	☐	For Bayesian analysis, information on the choice of priors and Markov chain Monte Carlo settings
☒	☐	For hierarchical and complex designs, identification of the appropriate level for tests and full reporting of outcomes
☐	☒	Estimates of effect sizes (e.g. Cohen's *d*, Pearson's *r*), indicating how they were calculated

Our web collection on statistics for biologists contains articles on many of the points above.

Software and code

Policy information about availability of computer code

Data collection	Cutadapt (v1.15) for Smart-seq reads processing and PISA (v0.7) for snRNA-seq and snATAC-seq reads processing.
Data analysis	R (v4.0.2), ArchR(v1.0.1) ,Seurat (v3.1.1), IGV□v2.10□, chromVAR (v.1.4.1) , cytoscape (v 3.9.1) , monocle2□v2.14.0□, metascape (https://metascape.org/gp/index.html), liftOver Signac (v1.6.0), motifmatchr□1.16.0□, chromVARmotifs(v0.2.0). Custom codes used in the analysis can be accessed use this github link:https://github.com/single-cell-BGI/MBA.

For manuscripts utilizing custom algorithms or software that are central to the research but not yet described in published literature, software must be made available to editors and reviewers. We strongly encourage code deposition in a community repository (e.g. GitHub). See the Nature Portfolio guidelines for submitting code & software for further information.

Data

Policy information about availability of data

All manuscripts must include a data availability statement. This statement should provide the following information, where applicable:

- Accession codes, unique identifiers, or web links for publicly available datasets
- A description of any restrictions on data availability
- For clinical datasets or third party data, please ensure that the statement adheres to our policy

The raw data generated in this study have been deposited in CNGB Nucleotide Sequence Archive (CNSA: https://db.cngb.org/cnsa) under accession code: CNP0000927. We have also provided the MBA website (https://db.cngb.org/mba), an open and interactive database for exploration. The public datasets used in this study can be accessed as described below: Allen Cell Types Database-Human Multiple Cortical Areas is available at https://portal.brain-map.org/atlases-and-data/rnaseq/human-multiple-cortical-areas-smart-seq. snATAC-seq data of adult human PFC is available at https://www.synapse.org/#!Synapse:syn22079621/. RNA ISH images for genes expressed in primary visual cortex of macaque brain is available at NIH Blueprint Non-Human Primate (NHP) Atlas: GFAP : http://

www.blueprintnhpatlas.org/ish/experiment/show/100140483, GPR83 : http://www.blueprintnhpatlas.org/ish/gene/show/183031, RORB : http://www.blueprintnhpatlas.org/ish/gene/show/183109, PDE1A: http://www.blueprintnhpatlas.org/ish/gene/show/183138 and SYT6 http://www.blueprintnhpatlas.org/ish/experiment/show/100091672. Summary statistics files for each human trait were downloaded from the UK Biobank database or published studies (data links in Supplementary Table 12). The JASPAR database (2020) for human TF motif is available at http://www.bioconductor.org/packages/release/data/annotation/html/JASPAR2020.html. Source data are provided with this paper.

Field-specific reporting

Please select the one below that is the best fit for your research. If you are not sure, read the appropriate sections before making your selection.

☒ Life sciences ☐ Behavioural & social sciences ☐ Ecological, evolutionary & environmental sciences

For a reference copy of the document with all sections, see nature.com/documents/nr-reporting-summary-flat.pdf

Life sciences study design

All studies must disclose on these points even when the disclosure is negative.

Sample size	Sample size was not per-determined. The minimum number of nuclei obtained from Smart-seq samples, droplet-based snRNA-seq samples and snATAC-seq samples are 415, 6896 and 8558, respectively. The sample size allowed us to obtain high coverage transcriptome and chromatin accessibility sites (peaks) for each cell type in each cortical region, and perform downstream analysis.
Data exclusions	For snRNA-seq and snATAC-seq analysis, we excluded nuclei based on quality control procedure. Due to the different levels of expression between SMART-seq2 data and DNBelab C4 drop-seq based snRNA-seq data and and fewer cell numbers of SMART-seq2 data (lack of cells in some EX- subtypes), we retained the DNBelab C4 RNA data for downstream analysis, including the inter-regional comparisons, peak-to-gene correlation, and disease risk enrichment, etc. For snATAC-seq data, we retained the cells with consistent cell type identified by snATAC-seq annotation and by snRNA-seq transferring.
Replication	We used three female 72-month-old cynomolgus monkeys for snRNA-seq and snATAC-seq, samples from all three cortical region (PFC, M1 and V1) were collected from each of the animals and processed for both snRNA-seq and snATAC-seq. All details appeared in Supplementary Table 1. Tissue sampled for Stereo-seq were from two female 60-month-old cynomolgus monkeys, three slices of PFC region and four slices of M1 region and two slices of V1 region were used for Stereo-seq. Findings in each modality were compared across biological replicates, we didn't observe any disagreement between replicates in terms of biological conclusion.
Randomization	No applicable because there is no group allocation.
Blinding	No applicable because there is no group allocation.

Reporting for specific materials, systems and methods

We require information from authors about some types of materials, experimental systems and methods used in many studies. Here, indicate whether each material, system or method listed is relevant to your study. If you are not sure if a list item applies to your research, read the appropriate section before selecting a response.

Materials & experimental systems

n/a	Involved in the study	
☐	☒	Antibodies
☒	☐	Eukaryotic cell lines
☒	☐	Palaeontology and archaeology
☐	☒	Animals and other organisms
☒	☐	Human research participants
☒	☐	Clinical data
☒	☐	Dual use research of concern

Methods

n/a	Involved in the study	
☒	☐	ChIP-seq
☒	☐	Flow cytometry
☒	☐	MRI-based neuroimaging

Antibodies

Antibodies used	Recombinant Alexa Fluor® 488 Anti-NeuN antibody [EPR12763] - Neuronal Marker (ab190195)<br>Recombinant Alexa Fluor® 488 Rabbit IgG, monoclonal [EPR25A] - Isotype Control (ab199091)
Validation	The validation by manufactures:<br>Alexa Fluor® 488 Rabbit monoclonal [EPR12763] to NeuN - Neuronal Marker (ab190195): https://www.abcam.com/alexa-fluor-488-neun-antibody-epr12763-neuronal-marker-ab190195.html<br>Alexa Fluor® 488 Rabbit IgG, monoclonal [EPR25A] - Isotype Control(ab199091): https://www.abcam.com/alexa-fluor-488-rabbit-igg-monoclonal-epr25a-isotype-control-ab199091.html

Animals and other organisms

Policy information about studies involving animals; ARRIVE guidelines recommended for reporting animal research

Laboratory animals	Three female 72-month-old and two female 60-month-old cynomolgus monkeys (Macaca fascicularis). To minimize the number of animals and excluded the effects of sex on limited number of biological replicates, we chose to use only one sex (female), which might be a limitation of the present findings without comparison between the female and male brain.
Wild animals	No wild animals were used in this study.
Field-collected samples	No field-collected samples were used in this study.
Ethics oversight	All relevant procedures involving animals were approved in advance by the Institutional Animal Care and Use Committee of Yunnan Key Laboratory of Primate Biomedical Research.

Note that full information on the approval of the study protocol must also be provided in the manuscript.
